# OrthoInspector: comprehensive orthology analysis and visual exploration

**DOI:** 10.1186/1471-2105-12-11

**Published:** 2011-01-10

**Authors:** Benjamin Linard, Julie D Thompson, Olivier Poch, Odile Lecompte

**Affiliations:** 1 Laboratoire de bioinformatique et genomique integratives, Département de Biologie et Génomique Structurales CNRS/INSERM/UDS, Institut de Génétique et de Biologie Moléculaire et Cellulaire, 1 rue Laurent Fries, 67404, Illkirch, Cedex, France

## Abstract

**Background:**

The accurate determination of orthology and inparalogy relationships is essential for comparative sequence analysis, functional gene annotation and evolutionary studies. Various methods have been developed based on either simple blast all-versus-all pairwise comparisons and/or time-consuming phylogenetic tree analyses.

**Results:**

We have developed OrthoInspector, a new software system incorporating an original algorithm for the rapid detection of orthology and inparalogy relations between different species. In comparisons with existing methods, OrthoInspector improves detection sensitivity, with a minimal loss of specificity. In addition, several visualization tools have been developed to facilitate in-depth studies based on these predictions. The software has been used to study the orthology/in-paralogy relationships for a large set of 940,855 protein sequences from 59 different eukaryotic species.

**Conclusion:**

OrthoInspector is a new software system for orthology/paralogy analysis. It is made available as an independent software suite that can be downloaded and installed for local use. Command line querying facilitates the integration of the software in high throughput processing pipelines and a graphical interface provides easy, intuitive access to results for the non-expert.

## Background

New sequencing technologies are dramatically increasing the number of predicted protein sequences available for high throughput comparative analyses, functional annotation or evolutionary studies. All these studies involve a transfer of information between organisms and homology is one of the most popular concepts used to address this problem. In particular, the studies rely on an accurate determination of orthology and paralogy relationships. According to the seminal definition of Fitch [1], orthologs are homologous genes that diverged from a single ancestral gene in their most recent common ancestor via a speciation event, whereas paralogs are homologs resulting from gene duplications. The distinction between orthologs and paralogs refers exclusively to the evolutionary history of genes and does not have functional implications *stricto sensu *[2]. However, from an operational point of view, it is widely accepted that two orthologs generally share the same function [3]. In contrast, paralogs are generally considered more divergent as new functions can emerge as the result of mutations or domain recombinations. Nevertheless, the multiplication of available genomes has underlined the necessity to distinguish two subtypes of paralogs: inparalogs and outparalogs [4]. Inparalogs are produced by duplication(s) subsequent to a given speciation event, while outparalogs result from an ancestral duplication (relative to the given speciation event). In other words, in-paralogy and out-paralogy are concepts relative to the species under comparison. The distinction is crucial in evolutionary studies since sets of inparalogs derive from orthologs by lineage-specific expansions and thus can be considered to be co-orthologs, while outparalogs do not have orthologous relationships at all.

Today, the most commonly used approach for the prediction of homology relationships between genes and proteins (and thus orthology and paralogy relationships) involves some kind of similarity measure, which can be linked to different types of data, such as sequences, domains or even 3 D structures. In principle, phylogenetic tree-based inference represents the most accurate way to determine orthology and paralogy [3-5]. However, its use at the complete proteome scale is computationally expensive and, given the rate at which new genomes are now being sequenced, cannot be considered as a viable option for most laboratories at the present time. As a consequence, alternative algorithms based on graphs or on a combination of tree and graph representations [6], have been developed to infer homology relationships. Most of them involve protein Blast all-versus-all searches and use pairwise distance calculations [7], 3-way best-hits [8-10] or clustering-based approaches [11-13]. In general, comparative studies [14,15] have shown that phylogenetic reconstructions have higher sensitivity and lower specificity than graph-based methods, particularly for distant organisms. Nevertheless, these methods provide good results for both sensitivity and specificity with some datasets [16,17]. However, each of the methods has advantages and disadvantages, and the most appropriate method will depend on the user's purpose [6,18]. Apart from the detection accuracy, other factors need to be taken into account, for example the availability and ease-of-use of the programs. Most of the methods commonly used today are made available as public software binaries and data browsing for the non-specialist is limited to web interfaces that allow remote querying of pre-calculated databases. For the more computer literate, large-scale queries can be performed and results can be retrieved in the form of flat files, although this requires a certain level of programming expertise to parse the data. To address this problem, some efforts have been made to facilitate the querying of data through presence/absence constraints and to provide global views of results via phylum-related tables [10]. Nevertheless, the tools are still available as web-based interfaces and cannot be retrieved locally to support or maintain in-house databases.

Here we describe OrthoInspector, a new software system incorporating an original algorithm for the rapid detection of orthology and in-paralogy relationships between different species. In comparisons with existing methods, it improves detection sensitivity, with a minimal loss of specificity. Moreover, OrthoInspector has a modular design and is provided as an independent software suite that can be downloaded and installed for local use. Command line querying facilities have been developed to allow fast information selection for high throughput studies and to facilitate the integration of the software in other packages or processing pipelines. An enhanced graphical interface is designed to automate the complete software installation and data generation process for non-specialists. Finally, different visualization tools have been designed specifically to allow the in-depth exploration of the complex inter-species orthology/in-paralogy relationships detected.

## Implementation

**Figure 1:**
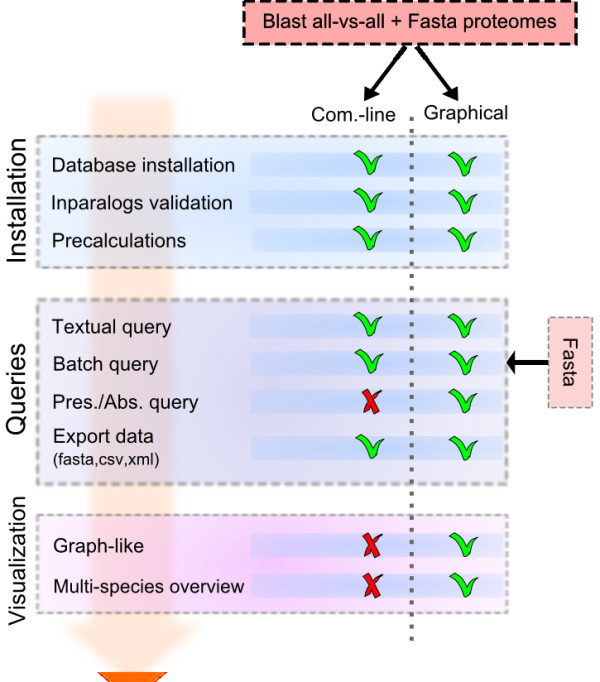
**OrthoInspector Suite overview**. OrthoInspector provides two user interfaces: a command-line client and a graphical interface. Installation operations include the creation of the database, the calculation of ortholog/inparalog groups and an optional creation of pre-calculated data. Queries include orthologous relationship searches, with or without advanced criteria: textual searches access results trough sequence accession numbers or sequence descriptions, batch queries allow submitting of multiple sequences in FASTA format and constraints of presence/absence of orthologs in specific organisms can be considered. Visualization tools provide different views for comparative studies.

The OrthoInspector suite is coded in Java 1.6.x, which means that it can be run on all Java-supporting platforms (UNIX, Windows, Mac....). Several java packages are incorporated: (i) the Jacksum package is used to encoded sequence data, (ii) the JDOM and opencsv packages are used to format sequence and orthology/paralogy data, (iii) the Jung and Prefuse packages are used to support the visualization tools. OrthoInspector also requires a background database to handle the huge amount of data produced by a Blast all-versus-all analysis. Support for the main "relational database" compatible engines (MySQL, PostgresSQL, Oracle...) is provided via definition of the corresponding java drivers in a configuration file. The only constraint is the predefined database schema that is needed by OrthoInspector. The software suite provides two different user interfaces, a command-line client and a graphical interface that can be used to perform the three steps involved in the complete analysis process (Figure 1):

1. Command-line and graphical versions can be used to perform Blast all-versus-all sequence searches and to generate a database containing the search results. Currently, the package is designed to allow the use of both raw and tabbed outputs produced by the classical NCBI Blast package and the recent NCBI blast+ package [19]. Other Blast data formats can be easily added with the help of the Blast parser interface included in the package. OrthoInspector proposes options (i) to directly fill the database with the produced data or (ii) to create intermediate data dumps allowing a considerable speed-up. Sql scripts to use these dumps in mySQL and postgresSQL engines are provided in the OrthoInspector website.

2. After database installation, the command-line version allows fast information retrieval for high throughput studies and the use of the software in other packages. Textual queries (accession numbers, description...), batch queries (Fasta sequences in a file) or queries defining presence/absence of an ortholog in specific organisms can be performed. Both command-line and graphical versions allow the user to export data in FASTA, CSV and XML formats. New output formats can be easily coded with the help of the output interface provided.

3. The graphical version facilitates data querying for non-specialists. In addition, it provides a set of useful tools to retrieve clusters of orthologs covering multiple species, to produce comparative genomics results and to visualize the data.

The whole software suite is available at http://lbgi.igbmc.fr/orthoinspector. Furthermore this website contains tutorials and database dumps for test purposes.

## Methods

### OrthoInspector algorithm

The OrthoInspector algorithm is divided into three main steps. First, the results of a Blast all-versus-all (proteomes are blasted against each other) is provided by the user and is parsed to find all the Blast best hits for each protein and to create the groups of inparalogs. Second, the inparalog groups for each organism are compared in a pairwise fashion to define potential orthologs and/or in-paralogs. Third, best hits that contradict the potential orthology between entities are detected.

### Inparalog group formation and validation

**Figure 2:**
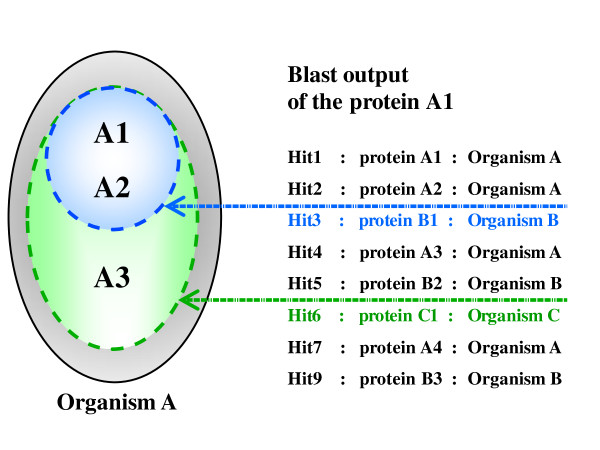
**Inparalog group formation and validation**. The hypothetical Blast search output for a protein A1 of the organism A. Two proteins of organism A are found with better scores than any protein of organism B: the protein A1 itself and the protein A2. If the blast output of A2 reproduces the same scenario, A1 and A2 are considered inparalogs with respect to organism B. Similarly, A1, A2 and A3 are inparalogs with respect to organism C, if these three proteins have a better score than any protein of organism C in the Blast results for A1, A2 and A3.

The first step involves the parsing of the Blast all-versus-all results to find all best hits for each protein and to create the groups of inparalogs, i.e. paralogs produced by duplications subsequent to a given speciation event (Figure 2). Inparalog groups are organism-dependant, which means that a given protein (*p*_n_) can be in different putative groups of inparalogs and we will denote these groups as organism-dependant lists: {*p1, p2, ..., pn*}^/organism^. Given a Blast search result for a protein of organism A, all proteins of A with an E-value inferior to the E-value of the best hit in the organism B will define a potential group of inparalogs in A with respect to the internal node where species A and B coalesce (we will refer to a group of inparalogs in A "with respect to B"). The putative list of inparalogs is then validated if the same minimal hypothesis of inparalogy is verified in the Blast searches for each protein in the list. As an example, we can consider a group of three putative inparalogs in organism A with respect to B (denoted {*A1, A2, A3*}^/B^) that has been defined by the Blast output of the protein *A1*. The entire group will be validated if the Blast outputs of *A2 *and *A3 *result in the same group. Thus, validation requires that the groups {*A2, A1, A3*}^/B ^or {*A2, A3, A1*}^/B ^are defined by the Blast output of *A2 *and that the groups {*A3, A1, A2*}^/B ^or {*A3, A2, A1*}^/B ^are defined by the Blast output of *A3*. If the above condition is not verified, the existence of two-member groups is checked. In the example, if the Blast output of *A1 *defines the group {*A1, A2, A3*}^/B ^but the Blast output of *A3 *defines a group of two proteins {*A3, A1*}^/B^, only this *A2*-deleted paralog group will be retained in the subsequent steps of the algorithm. Using this method, if n_orga _organisms are used to create the Blast all-versus-all, each Blast search can define n_group _< = n_orga _putative groups of inparalogs, each one being delimited by a best hit in another organism.

### Pairwise comparison of inparalog groups

**Figure 3:**
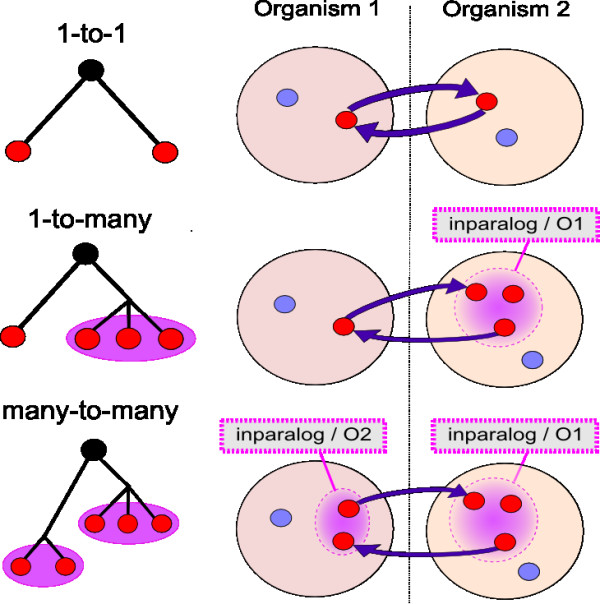
**Comparison of inparalog groups**. Blast best hits are used to define the potential relationships existing between inparalog groups. 1-to-1 relationships are equivalent to the classical reciprocal best hits (RBH). 1-to-many relationships are associated with potential duplication(s) after speciation in one of the lineages and cannot always be detected by RBH. Many-to-many relationships result from duplications in both lineages after speciation and again, cannot always be detected by RBH.

The second step of the OrthoInspector algorithm is the definition of potential (co)-orthology relationships (Figure 3). The definition is based on the detection of best hits existing between the two types of entities determined at the previous step: single proteins (not included in a group of inparalogs), and proteins belonging to one or several inparalog groups. We thus have three types of pairwise entity comparisons (*{protein <-> protein}*, *{protein <-> inparalogs} *and *{inparalogs <-> inparalogs}*), corresponding to the three types of relationships shown in Figure 3. A *1-to1 *relationship is described by a best hit between a protein of *O1 *and a protein of *O2 *complemented by a returning best hit from the protein of *O2 *to the protein of *O1, known as a reciprocal best hit*. A *1-to-many *relationship is described by a best hit from a given protein of *O1 *to any protein member of an inparalog group of *O2 *complemented by a returning best hit from any member of the inparalog group of *O2 *to the same protein of *O1*. Finally, a *many-to-many *relationship is described by two best hits between proteins of two groups of inparalogs (a group in *O1 *and a group in *O2*).

### Detection of contradicting information

The third step in the algorithm is the detection of best hits that contradict the potential orthology relationships defined above. In particular, given two inparalog groups that are potentially orthologous, it is possible to find a best hit from a protein in one of the compared groups to another protein that does not belong to either of the groups. In this case, it is possible that the protein does not belong to the inparalog group. Such contradictions are highlighted by OrthoInspector with a warning signal in the algorithm output: a "red signal" indicates contradictions involving a reciprocal best hit and an "orange signal" indicates contradictions involving a simple best hit. Such signals help the user to discriminate proteins in complex inparalog groups formed by closely related sequences or in cases where the proteome of one of the compared organisms is incomplete and disturbs the precedent formation of validated inparalog groups.

## Results

### Large-scale proteome analysis

We used the OrthoInspector software to study 59 organisms with approximately complete proteomes covering the main eukaryotic phyla in Protists, Fungi, Plants and Animals. We l incomplete and low coverage genomes to avoid predictions of false gene loss and artefacts in gene duplication inference [20]. The complete list of the 59 studied organisms with their taxonomic identifiers and the number of retained protein transcripts can be found in additional file 1. For 22 higher eukaryotes, protein sequence datasets from Ensembl 56 [21] were used. To avoid multiple transcript issues, the longest protein sequence was selected for each Ensembl-predicted gene annotated as 'protein-coding'. For example, the proteomes of *Homo sapiens *(22384 transcripts), *Mus musculus *(23117 transcripts), *Xenopus tropicalis *(18023 transcripts), *Ciona intestinalis *(14180 transcripts), *Arabidopsis thaliana *(31280 transcripts) or *Oryza sativa japonica *(57995 transcripts) were obtained from Ensembl. For eukaryotes not stored in Ensembl, the NCBI RefseqP [22] and Uniprot (Swissprot+TrEMBL) [23] databases were used. Data from both sources were retrieved using ICARUS scripts on a local SRS server [24] to select sequences according to their taxonomic identifiers. To remove redundant sequences, each sequence was compared to all others from the same organism using Blast. For sequences sharing more than 99% identity, manually-annotated entries from Swissprot were preferred over TrEMBL and RefseqP entries, otherwise the longest sequence was retained. Proteomes built with this protocol include *Plasmodium falciparum *(5234 transcripts), *Trypanosoma brucei *(8928 transcripts), *Ostreococcus tauri *(7974 transcripts), *Encephalitozoon cuniculi *(1903 transcripts), *Emericella nidulans *(9732 transcripts), *Saccharomyces cerevisiae *(6771 transcripts), *Laccaria bicolor *(17698 transcripts), *Caenorhabditis elegans *(22614 transcripts), *Ixodes scapularis *(21009 transcripts) and *Drosophila melanogaster *(22430 transcripts). Regardless of the source sequence database, sequences with less than 20 amino acids or more than 10000 amino acids were excluded. Finally, we obtained a pool of 940855 protein sequences.

The new NCBI-Blast+ package was then used to perform Blast all-versus-all searches between the proteomes of the 59 organisms, representing 940855 individual Blast searches in a database of 940855 sequences. Sequences were selected with an E-value cutoff of 1e-9. The searches were executed on the Décrypthon grid resources [25].

**Figure 4:**
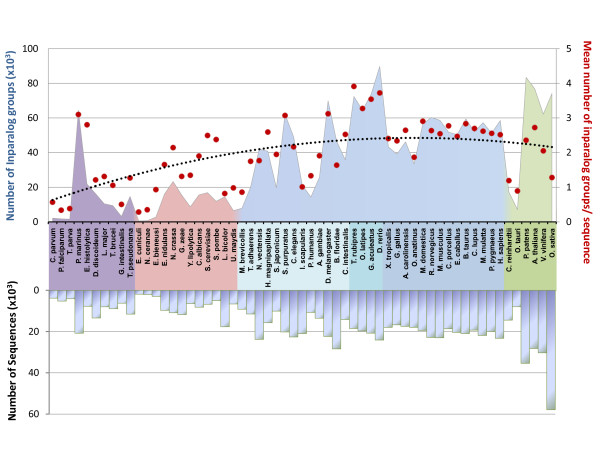
**Distribution of predicted inparalog groups over 59 organisms**. Organisms are ordered by their phylum and their decreasing number of inparalogs (green: viridiplantae, dark blue: tetrapoda, blue: teleostei, clear blue: other bilateria, pink: fungi, purple: other eukaryotic groups). The mean number of inparalog groups per sequence (red dots) is linked to the size of the proteomes (negative y axis). The black dotted line represents the corresponding polynomial tendency curve.

The results of the Blast all-versus-all searches, together with the 59 proteomes were then used as input to OrthoInspector. All steps of the algorithm, from Blast parsing to integration of the data in the relational database, took about 20 hours on four 2.67 GHz Intel Xeon CPUs with 6 Go of RAM. This timing is based on an installation of the database using the faster "database dumps" configuration (see Implementation). In more detail, parsing of the Blast results took 5h20, validation of inparalog groups took 2h10 and generation of 1-to-1, 1-to-many and many-many precalculated data for the 59 organisms took 12 h. The inparalog prediction step produced 10342157 putative inparalog groups, themselves generating 2073328 validated groups (Figure 4). Shortest versions of this huge dataset (> 100Go), including 7 proteomes, are available as database dumps (mySQL and postgresSQL) at the OrthoInspector website http://lbgi.igbmc.fr/orthoinspector.

As expected, large-scale proteomes, e.g. in plants (green color in Figure 4), or genome-wide duplications, e.g. in fishes (medium blue), result in an increase in the number of predicted inparalog groups, whereas smaller eukaryote proteomes have relatively few groups. The number of inparalog groups is generally correlated with the proteome size and the phylogenetic distance between organisms, for instance, amniota (dark blue) have a relatively stable number of inparalog groups. Nevertheless, some exceptions can be observed. Despite having the largest proteome in the plant phylum, Oryza sativa has fewer groups than Vitis vinifera or Arabidopsis thaliana and sequences from Oryza sativa are included in relatively few inparalog groups. Further investigation showed that many sequences of this organism had a relatively small number of Blast hits to other organisms compared to other plants (data not shown). This may be partly due to some overprediction of genes in the Oryza sativa proteome, with several protein fragments or pseudogenes predicted as "protein-coding".

Another interesting observation is that all parasitic organisms generate a small number of inparalog groups compared to the other members of their phylum. In arthropods, Ixodes scapularis (deer tick) and Pediculus humanus (body louse) have less inparalog groups than Anopheles gambiae and Drosophilia melanogaster. In fungi, Encephalitozoon cuniculi, Nosema ceranae and Ustilago maydis have less inparalog groups than other members of this phylum. Lacarria bicolor is another fungus with few inparalogs, although this may be linked to its ectomycorrhizal symbiotic relationship with plant roots.

Unlike parasites or symbionts, some isolated organisms have a relatively large number of inparalog groups. For example, Strogylocentrotus purpuratus has numerous inparalog groups but is currently the only echinodermata genome available, and it is impossible to determine whether this is a characteristic of this phylum. Entamoeba histolytica has a number of inparalog groups similar to that to other organisms with the same proteome size, but individual sequences are included in more inparalog groups compared to other organisms. This might be explained by the lower quality of the proteome and/or the presence of numerous repeats, resulting in multiple Blast hits in all studied species.

**Figure 5:**
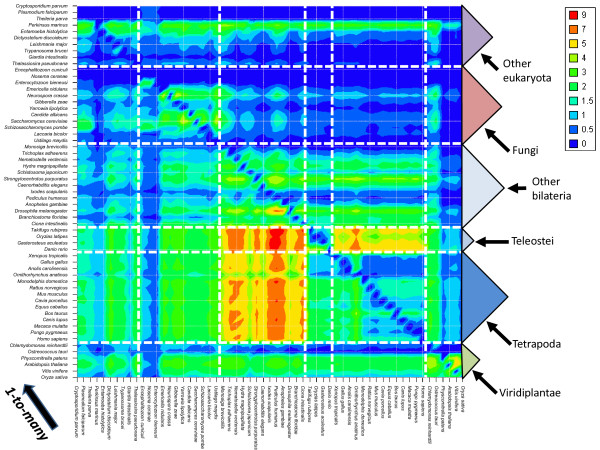
**Distribution of 1-to-many relations over 59 organisms**. The normalized number of 1-to-many relations is calculated for each organism pair. Normalisation is done by dividing the observed number of relations by the maximum number of potential relations (the size of the largest proteome of the two compared organisms). The 1-to-many relation is oriented from the x axis to the y axis.

In order to identify potential orthology relationships, all the inparalog groups were compared for each pair of organisms. The total number of relationships detected represents 8,649,287 1-to-1 relations, 2,648,403 1-to-many relations and 469,810 many-to-many relations. Figure 5 and additional files 2 and 3 show respectively the number of 1-to-many, 1-to-1 and many-to-many between each proteome pair after normalization. The number of predicted relationships is largely dependent on the composition of the set of selected organisms. As expected, close species present a high proportion of 1-to-1 relationships within their group but few many-to many relationships (additional files 2 and 3). This is especially obvious for the 18 vertebrates included in our dataset that are phylogenetically very close to each other compared to the other studied phyla. Intergroup relationships highlight lineage-specific duplications. For instance, the 2 whole genome duplications (WGD) encountered by the jawed vertebrates [26] are clearly reflected by the high number of 1-to-many relationships from invertebrates to vertebrates (Figure 5). Similarly, 1-to-many relationships pinpoint the additional round of duplication encountered by the teleostei lineage within vertebrates [27]. The numerous duplication events reported in the land plants [28] explain the extent of 1-to-many relationships between them and most of other species used in our study. Additionally, the abundance of many-to-many relationships between Physcomitrella patens (moss) [29]and flowering plants is in agreement with the independent events that occurred in the moss lineage (simple duplication) and hexaploidy event in flowering plants. Examination of specific sets of relationships (data not shown) is in agreement with dedicated studies. For instance, the functional analysis of the human genes exhibiting one-to-many relationships with rodents reveal a significant enrichment in gene related to olfaction as previously reported [30].

### Example test case: myotubularin family

**Figure 6:**
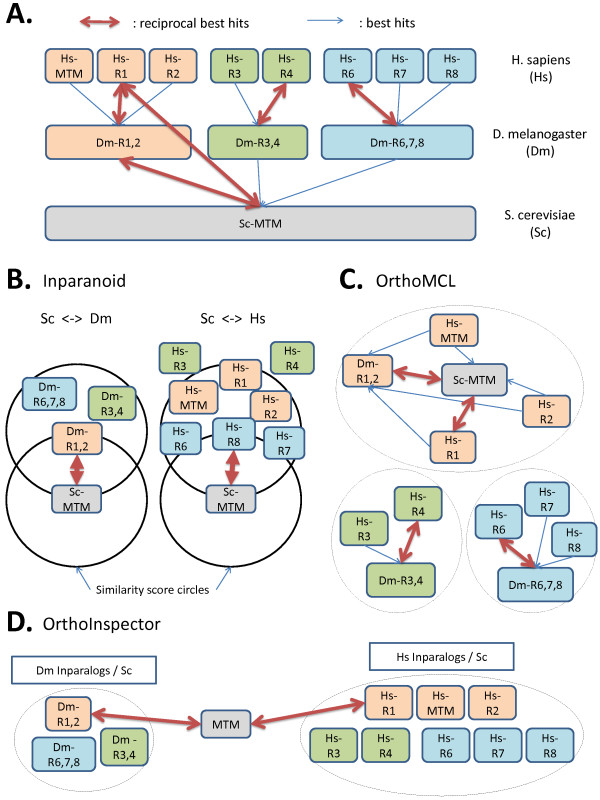
**Myotubularin family predictions**. A. The myotubularin family distribution is established in three species: *H. sapiens *(Hs), *D. melanogaster *(Dm) and *S. cerevisiae *(Sc), and multiple duplication events have been identified. Reciprocal best hits (RBH) and best hits (BH) linking the sequences are represented as red and blue arrows. Sequences used are Hs-MTM (MTM_HUMAN), Hs-R1 (MTMR1_HUMAN), Hs-R2 (MTMR2_HUMAN), Hs-R3 (MTMR3_HUMAN), Hs-R4 (MTMR4_HUMAN), Hs-R6 (MTMR6_HUMAN), Hs-R7 (MTMR7_HUMAN), Hs-R8 (MTMR8_HUMAN), Dm-R1,2 (Q9VMI9), Dm-R3,4 (Q7YU03), Dm-R6,7,8 (Q8MLR7), Sc-MTM (P47147). B. Inparanoid first identifies RBHs, then searches for putative inparalogs around these RBHs in a search space delimited by similarity score circles. The prediction between Hs and Sc excludes Hs-R3 and Hs-R4 sequences. C. OrthoMCL constructs a graph with proteins as nodes and similarities as edges, then identifies sub-graphs corresponding to co-ortholog groups. In this example, 5 human myotubularins and 2 fly myotubularins are excluded from the group containing the yeast myotubularin. D. OrthoInspector defines inparalog groups, then compares groups between organisms based on RBH and BH relations. Here, inparalog groups containing all the members of the myotubularin family are identified in human and fly, with no false negatives.

To demonstrate the advantages of using inparalog group comparisons to predict orthology, we studied the myotubularin family as a test case. The distribution of myotubularin-related proteins is well established [31] and is represented in Figure 6 for three species with multiple duplication events that occurred during its evolutionary history. OrthoInspector predictions are compared to Inparanoid and OrthoMCL, illustrating the algorithmic differences that lead to some false negatives for the two latter algorithms.

Inparanoid is based on RBH and finds inparalogs having a similarity score equal to or superior to the similarity S defined by the RBH. In the fly/yeast comparison, the three fly myotubularins are more similar to each other than to the yeast myotubularin, thus they are considered as inparalogs. In the human/yeast comparison case, 6 out of 8 human myotubularins have a higher similarity score than the similarity score defined by the yeast/human RBH, but 2 proteins have lower scores and are thus not considered as inparalogs (false negatives).

The OrthoMCL algorithm begins with the same steps of RBH detection and identification of sequences within the same genome that are more similar to each other than to any sequence from another genome. Then, a graph is constructed, where nodes represent proteins and edges represent the relations, and a Markov clustering is performed. In this example, three clusters are found, with only one fly and three human myotubularins considered to be co-orthologs of the yeast myotubularin.

OrthoInspector does not consider RBHs as a preliminary condition to detect potential inparalogs, instead inparalog groups are inferred directly in each organism. For example, the three fly and eight human myotubularins are identified as inparalogs with respect to yeast. In a second stage, the pairwise comparison of inparalog groups exploits the RBH and BH found between the different organisms to infer many-to-one relations including all the myotubularins.

### Comparison with existing methods: benchmark data sets

The accuracy of the OrthoInspector predictions was compared to five existing methods, covering the main approaches to infer orthology: namely, Inparanoid (pairwise distance comparisons), eggNOG (3-way best hits), OrthoMCL and OMA (graph clustering) and Ensembl compara (phylogenetic tree inference). Today, these methods are widely used by the community and their databases are cross-referenced in public databases like Uniprot. OrthoInspector is based on a pairwise distance based algorithm which makes it similar to the Inparanoid algorithm in some aspects. However, Inparanoid is directly based on reciprocal best hits (RBH) to find orthologs and inparalogs, as illustrated by the example test case described above. The first step of our algorithm identifies potential inparalog groups independently of RBH, thus exploring a larger search space for the discovery of potential orthology relations. The second step of our algorithm then compares inparalog groups that are not necessarily linked by a RBH between two organisms.

In order to compare the predictions made by OrthoInspector with the existing methods in a large scale study, we used two benchmarks from the literature [32,33], representing varied protein families (nuclear receptors, hox families, membrane receptors...). The literature benchmarks cover many organisms, including *H. sapiens, M. musculus, G. gallus, D. rerio, D. melanogaster, C. elegans *and *S. cerevisiae*. In addition, we created our own benchmark, performing a detailed study of protein kinase families with complex evolutionary histories that represent a significant challenge for the accurate detection of orthology/paralog relationships. Protein kinases represent an ideal test case for our purposes, since they have been intensively studied and their family relationships are generally known. In fact, protein kinases have been classified into a number of groups sharing broad functional properties, based on sequence similarity in their catalytic domains, the presence of accessory domains and known modes of regulation. Using the standard classification, available at http://kinase.com/kinbase, and by studying the literature, we defined a test set of well annotated protein kinase sequences, from the CMGC group (including cyclin-dependent kinases, mitogen-activated protein kinases, glycogen synthase kinases and CDK-like kinases) and from the TKL (tyrosine kinase-like) group. CMGC kinases represent a homogeneous group, where most proteins possess only the kinase catalytic domain. In contrast, the TKL kinases are more divergent, often having additional domains that regulate kinase activity, link to other signaling modules, or localize the protein in the cell. The CMGC and TKL groups can be further sub-divided into several protein families. The distribution of these families was established by a combination of published *in silico *and wet-lab studies in a number of model organisms, including *D. discoideum *[34], *C. elegans *[35], *S. cerevisiae *[36], *D. melanogaster *[37], *M. musculus *[38] and *H. sapiens *[39]. Our test set consisted of 329 manually annotated sequences from these six organisms, covering 31 CMGC sub-families and 16 TKL sub-families (additional file 4).

**Figure 7:**
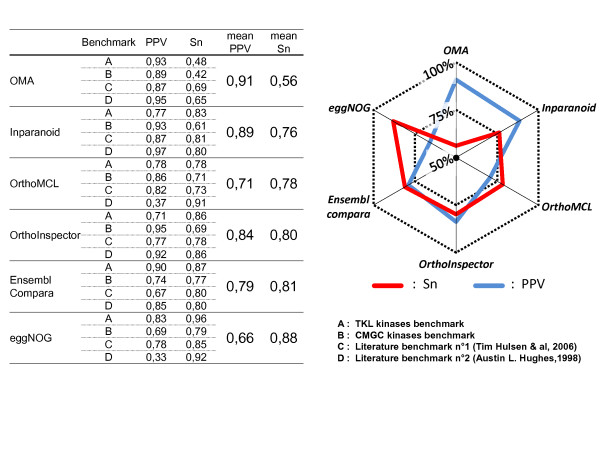
**Sensitivity and specificity comparison based on 4 benchmarks**. Two literature benchmarks and human CMGC and TKL kinases were used to evaluate the prediction accuracy for OrthoInspector and five other methods. Sensitivity (Sn) and Positive Predictive Values (PPV) were calculated for each method on each benchmark. The radar plot resumes the mean PPV (pink) and sensitivity (blue) for each method.

We then evaluated the predictions made by each of the six methods to the known classifications defined in the four benchmarks. The prediction accuracy was estimated by calculating the Positive Prediction Value (PPV) as a specificity indicator and the sensitivity (Sn) of each method (Figure 7). The benchmark data sets allowed us to highlight a number of advantages and disadvantages of the different methods. For example, OMA achieved the highest specificity, but the lowest sensitivity on average. In contrast, eggNOG obtained the highest sensitivity, although it should be noted that some co-ortholog groups in eggNOG are manually curated, like the COG database on which it is based. On average, the six methods can be classified in two groups. OrthoMCL, Ensembl compara and eggNOG have higher sensitivity than specificity, while Inparanoid, OrthoInspector and OMA have higher specificity than sensitivity. In the second class, OrthoInspector demonstrated higher sensitivity than the other two methods. In fact, OrthoInspector reached a sensitivity level close to that of Ensembl compara (80% and 81% respectively) and superior to OrthoMCL (78%).

Taken individually, the four benchmarks highlighted some contrasting results. For example, OMA obtained a sensitivity <50% for both TKL and CMGC benchmarks, compared to >60% for all other methods. This was due to the fact that OMA failed to predict some orthology relations existing between distant organisms (e.g. human and *C. elegans, S. cerevisiae *or *D. discoideum*). Ensembl compara had higher sensitivity than OrthoInspector for both kinase benchmarks (TKL:+1%, CMGC:+6%) and OrthoMCL had higher sensitivity for the CMGC kinases (+2%), but not TKL kinases (-8%). In the case of the literature benchmark n°1, all methods achieved a good sensitivity and a good specificity, which was not unexpected since the benchmark contains essentially human/mouse and human/worm relations. For the literature benchmark n°2, the results were more variable. OrthoMCL and eggNOG had high sensitivity (> 90%), but their specificity was surprisingly low (< 40%). In this benchmark, some protein families (heat shock proteins, collagens...) are totally included in a few or a single cluster. This observation is particularly true in the case of distant organism comparisons (human versus *C. elegans, S. cerevisiae*...).

It is clear from these results that the different methods tested here provide complementary approaches for orthology inference. In the future, it should be possible to combine the advantages of the alternative methods to improve both sensitivity and specificity. For example, OrthoInspector could be used as a starting tool to infer orthology relations, since its sensitivity and specificity are well balanced compared to most of the other methods tested here. Furthermore, the orthology inference is less computationally intensive than Ensembl compara, the only other method that achieved similar results. In a subsequent refinement step, the user could then integrate information about true/false positives from lower specificity methods such as eggNOG, OrthoMCL or Ensembl compara and lower sensitivity methods like Inparanoid or OMA methods.

### Data management and visualization

The main goal of the OrthoInspector project was to build a complete software suite for orthology and inparalogy prediction and analysis. Nevertheless, in the face of the huge amounts of data being produced by the new sequence technologies, it was clearly crucial to incorporate efficient data management and update procedures in the design of the software. Thus, the complete construction of a database of orthologs can be managed via a four step user-friendly process. OrthoInspector provides administrator tools, accessible via a command-line or a graphical interface, that take as input: (i) the results of a Blast all-versus-all search in a specified directory, (ii) the fasta proteomes of the organisms used in the Blast searches together, with an XML format file describing the organisms (name, source, taxonomic identifier...). The administrator can then launch the installation procedures that will automatically fill a database with all the required information and calculated data. Subsequent updates of the database are facilitated by the architecture of the database. For example, new proteomes can be added by updating the previously mentioned input data. In contrast to other available systems, after installation the pre-calculated data can be exploited via both command-line and graphical interfaces.

The command-line client interface is designed to allow fast information retrieval for high throughput studies. It also facilitates the incorporation of the software in other packages or processing pipelines. The client provides database querying facilities via a number of different methods: textual searches allow access to results via sequence accession numbers or sequence descriptions, while batch queries permit submission of multiple Fasta-formated sequences. In addition, constraints of presence/absence of orthologs in specified organisms can be defined. Data can be exported in CSV, FASTA or XML formats. New user-defined file formats can easily be added to the software using a java interface included in the source code.

**Figure 8:**
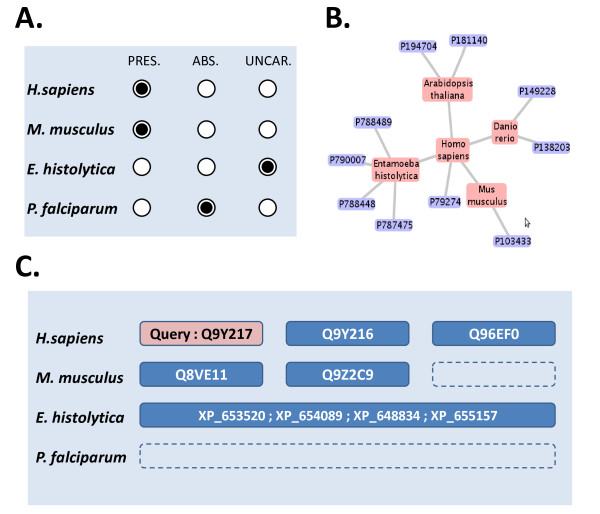
**OrthoInspector graphical interface**. The graphical interface provides visualization tools allowing a global view of the selected data. A. The advanced query interface allows selection of orthology/inparalogy relationships based on presence/absence criteria (pres. = presence, abs. = absence, uncar. = uncaring). B. Graph-based visualization of selected relationships. C. Presence/absence diagrams resume the repartition of orthologs/inparalogs for a family of proteins. Here, the human myotubularin-related protein 6 (mtmr6, Q9Y217) was used as the query. No orthology relationship is found in *P. falciparum*, a 1-to-1 ortholog is found in *M. musculus *(Q8VE11) and a 1-to-many relationship involving four co-orthologs is found in *E. hystolitica *(XP_653520; XP_654089; XP_648834; XP_655157). These sequences are then used as query to find potentially new sequences of the family in these organisms. Here sequences of E. histolytica make a 1-to-many relation with the seven human myotubularins and the six mouse myotubularins (these ones are inparalogs relative to entamoeba histolytica). Here are only represented the human MTMR7 (Q9Y216) and MTMR8 (Q96EF0) and the murine MTMR7 (Q9Z2C9).

The graphical interface is designed to analyze smaller sets of sequences in more detail. In contrast to the command-line client, the querying functions (textual and FASTA sequence queries) are supported by interactive forms and produce results that can be visualized in more detail. More elaborate queries can also be performed, such as the selection of data according to the presence/absence of orthologous relationships in organisms specified by the user (Figure 8A). For instance, the user can retrieve all *Danio Rerio *proteins having orthologs in *Homo sapiens*, but not in *Mus musculus*. The results can be visualized through a textual description, including cross-references to Ensembl, Uniprot and NCBI-refseqp databases. For ambiguous results, the original Blast search used to generate the prediction can be directly visualized in the interface. Then, the reliable data selected by the user can be summarized using different visualization tools. Currently, two complementary tools are available: (i) a graph representation of the network of predicted relationships (Figure 8B) and (ii) presence/absence diagrams (Figure 8C), but future updates of the software are planned to enhance the visualization capabilities of the software. As in the command-line client, the data can be exported in CSV, FASTA and XML format files. All the visualizations can be exported as image files, the presence/absence diagram can be exported as a CSV matrix and the graph representation can be saved in graphML format. The graphical interface access provides access to other tools, such as batch generation and exportation of data, generation of database statistics or switching between different OrthoInspector compliant databases.

## Conclusions

Various methods have been developed previously to predict the orthology/inparalogy relationships existing between different proteomes. In most cases, the algorithms are made publicly available in the form of binary programs that can generate either simple databases or flat files containing the complete set of predicted relationships. Until now, no comprehensive set of tools has been provided to process, query and update the datasets easily and efficiently. For this reason, we have developed OrthoInspector, incorporating fast and easy-to-use data management tools, as well as a novel algorithm to produce fast and sensitive predictions of orthology/inparalogy. The software suite, portable to any Java-compatible system and easily integrated in any workflow application, is suitable for use in high-throughput studies, which are becoming more and more predominant in the era of systems biology. Its fast and user-friendly procedures facilitate the production of databases adapted to the user's needs. It also supports more detailed analyses of interesting orthology relationships for non-specialists, who can exploit the generated databases in a graphical interface that provides novel visualization capabilities and comparative genomics tools.

In the future, OrthoInspector will be enhanced to further improve the database update process. Although tools are currently provided to easily incorporate new genomes selected by the user, keeping up with the rate of next generation sequencing will be a major challenge. The most time-consuming step in all orthology prediction algorithms is the generation of the Blast all-versus-all searches for each new update. In spite of the efforts aimed at developing faster parallelized Blast methods [40,41], the Blast all-versus-all computational requirements grow quadratically with the addition of new proteomes. Therefore, one of our future goals will be to develop an incremental update process, minimizing the number of distance calculations required between the thousands of sequences present in the previous version of the database. We also plan to enrich the OrthoInspector system by incorporating functional annotations, such as Gene Ontology terms [42] or links to the Interpro protein domain database [43], facilitating integrated systems biology studies. Finally, to improve the interoperability of OrthoInspector with other software packages, the Ortho-XML format http://orthoxml.org will be included in the next release of OrthoInspector.

## Availability and Requirements

Project name: OrthoInspector

Project home page: http://lbgi.igbmc.fr/orthoinspector/

Operating system: cross-platform

Programming language: Java

Requirements: Java JVM 1.6.x

License: GNU GPL version 3

## Authors' contributions

LB carried out the algorithm conception and the software programming. JDT participated in the design and testing of the software suite. OP participated in its design and helped to draft the manuscript. OL conceived the study and participated in its design and coordination. All authors participated in writing the manuscript. All authors read and approved the final version of the manuscript

## Supplementary Material

Additional file 1 **The complete list of the 59 studied organisms**. Excel file containing the 59 studied organisms in OrthoInspector. They are classified according to their phylum. Click here for file

Additional file 2 **Distribution of 1-to-1 relations over 59 organisms**. The normalized number of 1-to-1 relations is calculated for each organism pair. Normalisation is done by dividing the observed number of relations by the maximum number of potential relations (the size of the smallest proteome of the two compared organisms). Click here for file

Additional file 3 **Distribution of many-to-many relations over 59 organisms**. The normalized number of many-to-many relations is calculated for each organism pair. Normalisation is done by dividing the observed number of relations by the maximum number of potential relations (the multiplication of the size of the proteomes of the two compared organisms). Click here for file

Additional file 4 **Test set covering 31 CMGC sub-families and 16 TKL sub-families**. Excel file describing the 31 CMGC sub-families and 16 TKL sub-families used for benchmarking. Orthology predictions made by all methods for these families are in the file too. Click here for file
