# Supplementary figures and images for: OrthoInspector: comprehensive orthology analysis and visual exploration
Source: BMC Bioinformatics. 2011 Jan 10;12:11. doi: 10.1186/1471-2105-12-11 (PMC3024942; doi:10.1186/1471-2105-12-11)

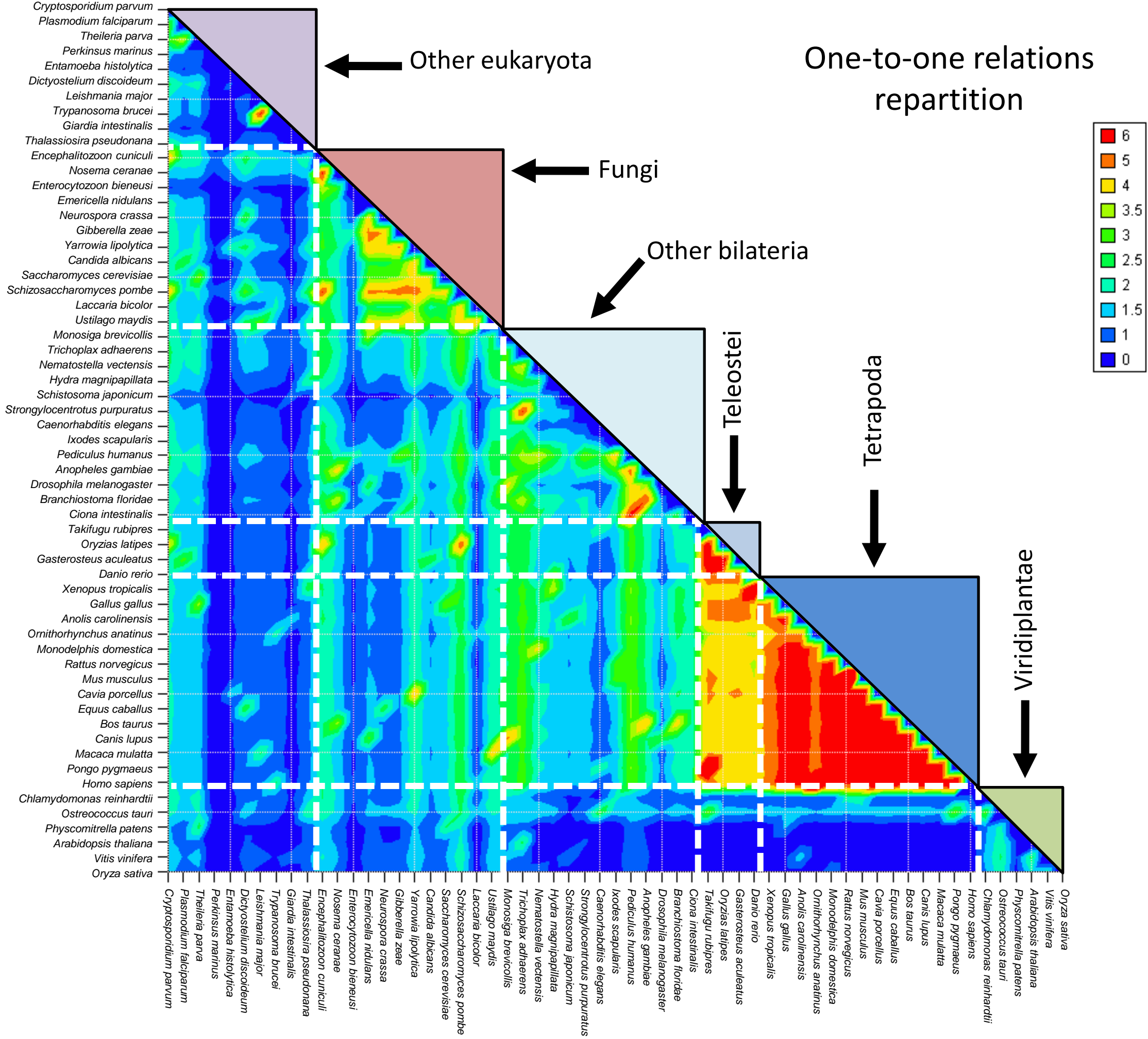

Supplement: Additional file 2 — Distribution of 1-to-1 relations over 59 organisms. The normalized number of 1-to-1 relations is calculated for each organism pair. Normalisation is done by dividing the observed number of relations by the maximum number of potential relations (the size of the smallest proteome of the two compared organisms). [file 1471-2105-12-11-S2.PDF]

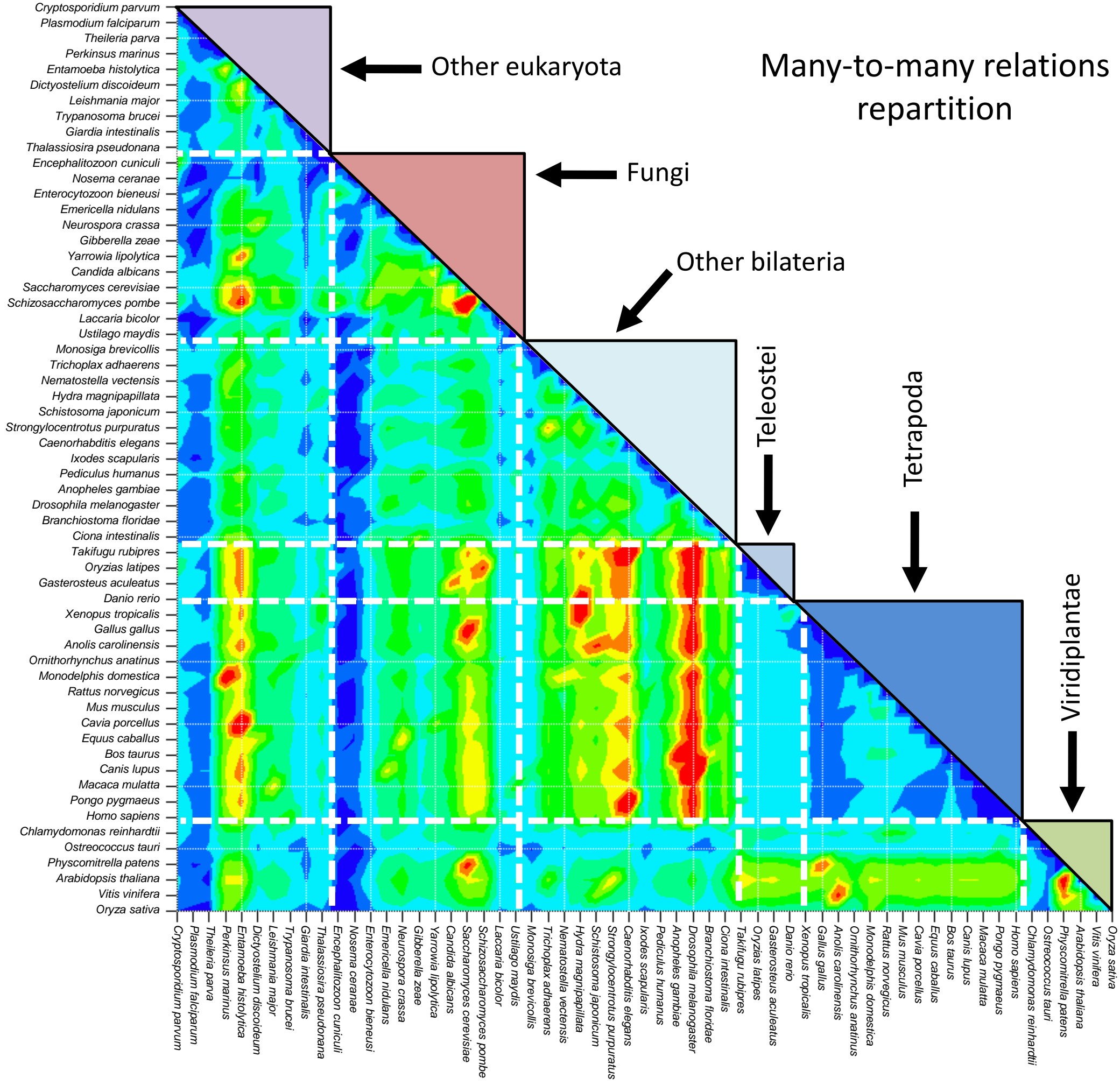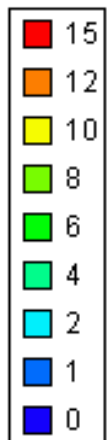

Supplement: Additional file 3 — Distribution of many-to-many relations over 59 organisms. The normalized number of many-to-many relations is calculated for each organism pair. Normalisation is done by dividing the observed number of relations by the maximum number of potential relations (the multiplication of the size of the proteomes of the two compared organisms). [file 1471-2105-12-11-S3.PDF]
